# Charge-Reversal Nano-Drug Delivery Systems in the Tumor Microenvironment: Mechanisms, Challenges, and Therapeutic Applications

**DOI:** 10.3390/ijms25189779

**Published:** 2024-09-10

**Authors:** Yizhu Liang, Jiashuai Wu, Yutong Yan, Yunduan Wang, Hongtu Zhao, Xiaopeng Wang, Shijie Chang, Shuo Li

**Affiliations:** 1 Innovation Institute, China Medical University, Shenyang 110122, China; 2022318102@cmu.edu.cn (Y.L.); 2022318101@cmu.edu.cn (J.W.); 2022318211@cmu.edu.cn (H.Z.); 2022318105@cmu.edu.cn (X.W.); 2 Department of Biochemistry & Molecular Biology, School of Life Sciences, China Medical University, Shenyang 110122, China; 2022353308@cmu.edu.cn; 3 Department of Biomedical Engineering, China Medical University, Shenyang 110122, China; wangyunduan@cmu.edu.cn

**Keywords:** charge-reversal nano-drug delivery systems, tumor microenvironment, stimuli-responsive, cancer-specific chemotherapy

## Abstract

The charge-reversal nano-drug delivery system (CRNDDS) is a promising system for delivering chemotherapy drugs and has gained widespread application in cancer treatment. In this review, we summarize the recent advancements in CRNDDSs in terms of cancer treatment. We also delve into the charge-reversal mechanism of the CRNDDSs, focusing on the acid-responsive, redox-responsive, and enzyme-responsive mechanisms. This study elucidates how these systems undergo charge transitions in response to specific microenvironmental stimuli commonly found in tumor tissues. Furthermore, this review explores the pivotal role of CRNDDSs in tumor diagnosis and treatment, and their potential limitations. By leveraging the unique physiological characteristics of tumors, such as the acidic pH, specific redox potential, and specific enzyme activity, these systems demonstrate enhanced accumulation and penetration at tumor sites, resulting in improved therapeutic efficacy and diagnostic accuracy. The implications of this review highlight the potential of charge-reversal drug delivery systems as a novel and targeted strategy for cancer therapy and diagnosis.

## 1. Introduction

Cancer is a major public health issue, accounting for nearly one in six deaths (16.8%) worldwide [1]. Among those who die prematurely from noncommunicable diseases (NCDs), 3 out of 10 succumb to cancer [2]. This underscores the importance of developing effective chemotherapy drugs and precise drug delivery systems. The commonly used chemotherapy drugs include paclitaxel, cisplatin, etoposide, etc. However, the low selectivity of these drugs often results in a series of side effects, including vomiting, hair loss, irreversible cardiotoxicity, and drug-induced leukemia, particularly with doxorubicin [3]. Additionally, paclitaxel also causes peripheral neuropathy as a common side effect [4,5].

Recently, the nano-drug delivery system (NDDS) has emerged a promising approach for drug delivery, specifically to the tumor microenvironment (TME) [6]. The NDDS, including inorganic nanoparticles, organic nanoparticles, and metal nanoparticles, often exhibits superior biocompatibility, precise targeting ability, higher bioavailability, and low cytotoxicity [7]. These systems have demonstrated significant benefits in terms of drug delivery, including applications in imaging, diagnosis, vaccine development, and treatment [8].

The NDDS plays a critical role in cancer treatment by effectively reaching the TME or being internalized by tumor cells. Previous studies have demonstrated that the surface charge of nanocarriers significantly influences the drug delivery efficiency and bioavailability [9,10]. The electric charge affects the cellular uptake through both direct and indirect mechanisms. Firstly, the isoelectric point of human serum albumin (HSA) is 4.7, causing it to present a negative charge in human blood, where the pH ranges from 7.35 to 7.45. Consequently, nanocarriers with positive points can electrostatically bind to these negatively charged plasma proteins, affecting the cellular uptake process. In contrast, the nanocarriers with a negative charge can maintain a longer blood circulation time without binding to the proteins, thus avoiding clearance by the mononuclear phagocytic system. Secondly, cationic nanoparticles have better cellular uptake ability and permeability than neutral or anionic nanoparticles [11,12]. To balance the higher drug concentrations in the blood with better cellular uptake efficiency, charge-reversal nano-drug delivery systems (CRNDDSs) were developed. The CRNDDS refers to a series of nanoparticles that combine both positive and negative charges. These systems provide both efficient cellular uptake levels and certain specificity against tumor.

Scientists have discovered several distinctive characteristics of the TME. One notable feature is the lower pH value, primarily caused by the lactic acid produced during aerobic glycolysis in tumor cells, which serves as a key energy source for tumor cells [13,14,15]. The acidic conditions in the TME also provide a favorable growth environment for tumor cells, promoting angiogenesis, metastasis, immune suppression, etc. [16]. Additionally, the TME is characterized by the presence of various enzymes that are highly expressed compared to their low or non-expression in normal cells or tissues. These specifically expressed enzymes have attracted attention for their potential in developing stimulus-responsive NDDSs triggered by the enzymes, which has received attention from researchers, for they have good specificity and mild reaction conditions [17,18]. Previous studies have also identified the disrupted redox homeostasis in tumor cells, such as the increase in reactive oxygen species (ROS) and H2S [19]. Based on these TME features, various CRNDDSs with different stimulus responses have been developed, which will be detailed in the following sections.

## 2. Charge-Reversal Nano-Drug Delivery System

The CRNDDS can release its loading drugs in response to specific stimuli that alter the charge on the surface. They not only prolong the circulation time of drugs in the blood but also enhance the efficiency of cellular drugs uptake. When the CRNDDS enters the TME, the surface charge of the drug delivery system usually changes from negative to positive [20,21]. This transformation is usually triggered by specific factors, and based on the triggering conditions, the CRNDDSs can be categorized into pH, enzyme, redox, NaCl, ATP, light, and thermal-responsive charge reversal systems.

### 2.1. pH-Responsive Charge-Reversal Systems

**Figure 1:**
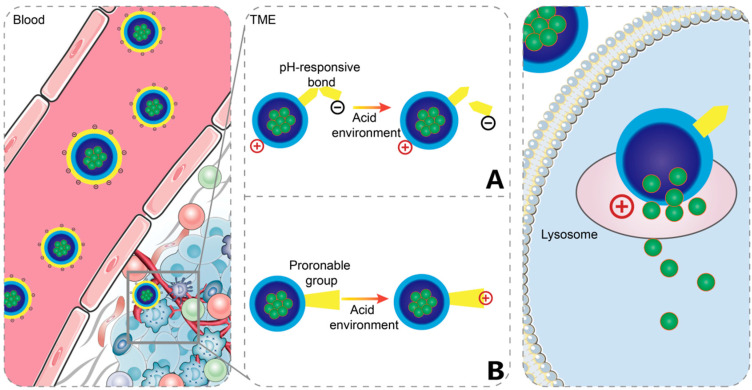
Two mechanisms (as shown in sub-image **A** and **B**) to achieve a positive charge reversal in acid-responsive charge-reversal systems: cleavage of pH-responsive bonds or protonation. (Figure 1 was created with Blender 4.2.0).

The production of lactic acid through aerobic glycolysis and the cleavage of ATP to release protons in tumor tissue result in a lower pH within the TME [22]. Measurements indicate that the pH range of most solid tumor patients varies from 5.7 to 7.8, compared to the normal blood pH of 7.4 [23]. The pH-responsive charge-reversal system occupies the vast majority of responsive charge-reversal systems. Its charge conversion methods can be categorized into two types: cleavage of chemical bonds and protonation and deprotonation of functional groups. Figure 1 below illustrates the mechanism of charge reversal.

#### 2.1.1. Cleavage of Chemical Bonds

Many chemical bonds have been designed to remain stable in neutral environments and cleave in acidic environments. These bonds could exist in the main or the side chains of drug delivery systems, or between positively charged cores and negatively charged shells. In the latter case, the hydrolysis of the outer shell upon entering the TME exposes the positively charged core, achieving charge reversal. The known acid-labile chemical bonds include amide bonds, ester bonds, acetal linkage, oxime bonds, imine bonds, etc. [24].

Amide bonds are the most common chemical bonds and β-carboxylic amide bond is widely applied in recent drug delivery systems. 2,3-dimethylaleic anhydride (DMMA) is often used to prepare these acid-sensitive chemical bonds, reacting with amino groups to form β-carboxylamide bonds [25]. Meng et al. developed a micelle nanosystem comprising two parts: a pH-responsive detachable polyethylene glycol-polylysine-dimethylmaleic anhydride (PEG–PLL–DMMA, abbreviated as PPD) shell and an MA-conjugated CpG-condensed PEI polycation core (MA-PEI-CpG, abbreviated as MPC). After exposure to a pH 6.8 environment, the surface potential reversed from −7.82 to +23.29 mV, and the chemical migration of hydrogen adjacent to the amide bonds/amion group in the PDD/MPC nanosystems was discovered through 1H NMR analysis. This change demonstrated that the hydrolysis of the DMMA amide bond in the PDD shell under acidic conditions leads to a switch in the surface potential [26]. Similarly, Zhu et al. utilized DMMA to protect nanoparticles from being cleared by the reticuloendothelial system, modifying their NPs to obtain Fe_3_O_4_&Sor@MSN-DMMA (F&SMD) [27]. Under acidic conditions, the 2,3-dimethylmaleic acid amide bond between the DMMA and NPs was broken, exposing the original positive potential [28].

Schiff bases are mainly a class of compounds containing C=N groups. These structures are unstable under acidic conditions and readily undergo hydrolysis [29]. Leveraging its properties, polymer nanodelivery systems incorporating Schiff bases can be designed to be acid-sensitive, facilitating acid-responsive charge reversal. Chen et al. developed a pH-responsive charge-convertible drug delivery nanocarrier (MSN-TPZ-GOx@ZnO@PAH-PEG-DMMA, abbreviated as MTGZ@PPD). The nanocarrier maintained a negative charge in a pH 7.4 environment; however, after incubation at pH 6.5 for two hours, the Schiff base structural bonds in the MTG hydrolyzed, and the β-carboxamide bonds in the PPD also underwent hydrolysis, resulting in a positive surface charge transformation. MTGZ@PPD has been developed for the synergistic treatment of hunger and chemotherapy, achieving efficient intracellular internalization and controllable drug release [30]. Tao et al. designed an acid/reduction dual-sensitive nano micelle, FA-PUSS-gimi-mPEG, which underwent the cleavage of benzoic-imine bonds under slightly acidic conditions. This cleavage caused the detachment of its hydrophilic layer and exposed the folic acid, achieving a reversal of the surface charge from negative to positive. Additionally, the disulfide bonds in polymers contribute to the micelles’ reduction sensitivity in high concentrations of L-glutathione (GSH) [31].

Borate ester bonds represent another type of acid-labile chemical bond. Researchers have designed a microenvironmental targeted nanoplatform using the cleavage of borate ester bonds for the treatment of pneumonia caused by *Pseudomonas aeruginosa* [32]. Fang et al. synthesized a covalent polyplex consisting of oHA-PBA and DHPA-CDB/Cur (oHA-PBA@DHPA-CDB/Cur). The borate ester bonds cleaved in a mildly acidic tumor microenvironment and exposed the cationic micelles, achieving a charge reversal from −19.47 to +12.01 mV. This nanocarrier exhibits good mitochondrial targeting capability and enhanced cellular uptake, making it a promising subcellular specific drug delivery system [33]. Another study involved synthesizing a positively charged polymer comprising gallic acid-chitosan oligosaccharide-dithiopropionate acid-berberine (GA-CDB). The positively charged core GA-CDB@Cur and the negatively charged shell AS-PBA are connected via borate ester bonds, which break upon reaching the TME. This polysaccharide-based nanosystem enables mitochondrial targeting and lysosomes escape in tumor cells [34]. Given the ease of targeting of the mitochondria with lipophilic cations, future CRNDDSs could incorporate more mitochondrial-targeting agents for precise tumor eradication. Additionally, organelle targeting may emerge as a new direction for CRNDDSs, fostering the development of drug delivery systems aimed at specific organelles.

Orthoester linkages are also acid-sensitive bonds. Previous studies have demonstrated that DOX-loaded micelles with orthoester linkages exhibit pH-sensitive disintegration behavior, unlike DOX-loaded micelles without orthoester linkages, which can be applied to selective drug delivery [35]. Hu et al. developed metal–organic framework (MOF)-based polymer-coated hybrid nanoparticles, MOF@polymer. The polymer shell decomposed under acidic conditions due to the changes in the chemical structure under different pH levels, specifically the cleavage of the orthoester linkages. This process exposed the positively charged MOF core, facilitating pH-triggered charge reversal and drug release [36]. This new hybrid nanocarrier achieves effective accumulation of chemotherapy drugs in solid tumors and minimizes systemic toxicity.

#### 2.1.2. Protonation and Deprotonation of Functional Groups

Certain functional groups (such as amino, carboxyl, amine, etc.) can be introduced into nanomaterials to accept or donate protons, thereby achieving protonation and deprotonation. This process induces changes in the surface zeta potential and does not involve chemical bond cleavage. It offers a faster response to pH variations compared to systems containing acid-unstable bonds, which typically take about 1.5 h [37].

Carboxyl groups are common functional groups used in CRNDDSs. These groups remain subject to deprotonation in neutral or alkaline environments, while protonation occurs when entering acidic environments, resulting in a positive charge on the polymer surface and promoting their penetration into biofilms [38]. Consequently, polymers and nanocarriers containing carboxyl groups are well suited for the treatment of tumors and infections, both of which often involve acidic environments. Common polymers containing carboxyl groups include polyacrylic acid (PAA), poly ethylacrylic acid (PEAA), and polyhistidine (PHis), etc. For instance, Pham et al. prepared a diblock copolymer (P(VBTAC/NaSS)_17_-b-PAPTAC_50_; P(VS)_17_A_50_), which exhibits both pH and thermal responsiveness. Its pH-responsive behavior results from protonation and the pendant carboxyl groups of PAAc_49_, with its thermo-responsive behavior affected by the pH of the solution [39]. Many studies have demonstrated that PAAs can accept and donate protons to prepare pH-responsive materials for encapsulating chemotherapy drugs for tumor treatment [40,41].

Tertiary amine groups also play an important role in pH-sensitive cationic polymers due to their ability to bind protons under acidic conditions and release protons under alkaline conditions. There are two common types of polymers containing tertiary amine groups: PDEAEM and PBAE. PDEAEM polymers have tertiary amine groups in their side chains; however, due to the less ideal sensitivity to slightly acidic environments, these polymers are rarely used in tumor treatment [42]. PBAE, on the other hand, is synthesized using primary or secondary amine with diacrylate. Shi et al. fabricated a nanoplatform (TPL/PBAETK@GA NPs) via the host–guest interaction between the glycyrrhetinic-acid-modified poly(ethylene glycol)-adamantanecarboxylic acid moiety and the reactive oxygen species (ROS)/pH cascade-responsive copolymer poly(β-amino esters)-thioketal (TK)-β-cyclodextrin. At pH 6.5, which is lower than the pKb value of tertiary amine, the tertiary amine on the PBAE copolymer undergoes protonation and externalization toward the shell, causing the zeta potential of the NPs to change from −2.8 mV to above +15.4 mV [43]. Badparvar et al. synthesized polymeric nanoparticles based on disulfide-containing hyperbranched MeO-PEG-b-(NIPAAm-co-PBAE). Upon reaching the TME, the charge shifted to positive due to the protonation of the tertiary amine on the PBAE under the mild acidic conditions [44]. Tertiary amine groups are excellent pH-responsive functional groups, and nanocarriers containing tertiary amine groups have broad prospects for targeted drug delivery to tumor cells [45,46].

There are also studies on the application of polymers containing sulfonamide groups or imidazole groups in CRNDDSs. Chen et al. designed a liposomal system (PSD/DOX/Cypate-BTSL), where poly(methacryloyl sulfadimethoxine) (PSD) acted as a tumor extracellular pH-sensitive polymer, and PSD underwent deshielding at pH 6.5, resulting in the exposure of the cationic surface of the liposome [47]. Jia et al. developed a nanoparticle wrapped with polyethylene glycol-histidine (PEG-His) (PEG-His@BPC). The PEG-His@BPC was negatively charged, providing a longer blood circulation time. After reaching the TME, the weakly acidic conditions caused the protonation of histidine, resulting in the detachment of the PEG shell and exposure of the positively charged BPC, thereby achieving strong tumor penetration [48]

### 2.2. Redox-Sensitive Charge-Reversal Systems

**Figure 2:**
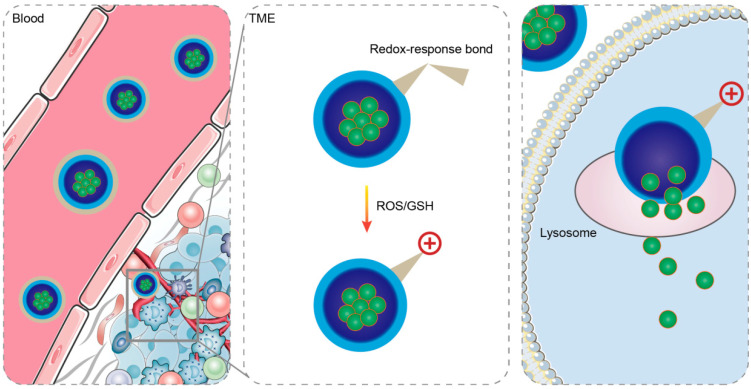
In the TME, the redox-sensitive charge-reversal system cleaves the redox-sensitive bonds, resulting in the entire system being positively charged. (Figure 2 was created with Blender 4.2.0).

The TME is characterized by different redox states compared to normal physiological tissues, including higher concentrations of GSH and increased levels of ROS [49,50]. These unique conditions provide triggers for charge reversal in drug delivery systems. Figure 2 demonstrates redox-sensitive charge-reversal systems’ working principle.

#### 2.2.1. ROS-Sensitive Charge-Reversal System

Tumor patients often experience disturbances in their redox homeostasis, leading to excessive production and accumulation of ROS [51,52]. Common ROS include hydroxyl radicals (OH·), superoxides (O^2−^·), nitric oxide (NO·) and hydrogen peroxides (H_2_O_2_) [53]. Zhang et al. reported an ROS-responsive Fe_3_O_4_-based nanoparticle capable of undergoing charge reversal and disassemble under overexpressed H_2_O_2_ in the TME. This capability for ROS-responsive disassembly was conferred by the ligand 3,3′-(Propane-2,2-diylbis (sulfanediyl)) dipropionic acid [54]. Kuang et al. designed cationic chitosan–drug conjugates employing an ROS-responsive aromatic thioacetal linker. At the lesion site, high concentrations of ROS caused the thioacetal junction to undergo in situ cleavage, releasing the coupled drugs [55]. Previous studies have also reported ROS-unstable polymers, including poly(propylene sulfide), selenium-containing polymers, poly(L-methionine), etc. [56]. These findings provide research ideas for using these polymeric nanocarriers to prepare ROS-sensitive CRNDDSs for the treatment of tumors.

#### 2.2.2. GSH-Sensitive Charge-Reversal System

Research has found that the level of GSH rises in various cancer, including brain, breast, gastrointestinal, gynecological, head and neck, and lung cancer. GSH plays a role in tumor occurrence, proliferation, and metastasis [57,58]. Under the action of GSH, disulfide bonds can be cleaved, a characteristic that can be used to develop GSH-sensitive CRNDDSs. Xu et al. constructed a nanosystem named DA-ss-DT, which conjugated 3,3′-dithiodipropionic acid-modified doxorubicin (DTPA-DOX) and 2,3-dimethylmaleic anhydride (DMA) to the amino groups of poly(ethylene glycol)-b-poly(L-lysine) (PEG-b-PLL) to produce the pH-sensitive and redox-sensitive part: PEG-b-P((LL-g-ss-DOX)-(LL-g-DMA)), and then encapsulated the triptolide (TRI) into the polymer micelles. In the bloodstream, the DA-ss-DT nanoparticles maintained a negative charge. Upon reaching tumor tissue, the cleavage of the amide bonds in DMA under acidic conditions led to a positive charge transformation, facilitating uptake by tumor cells. Subsequently, the high concentrations of GSH in tumor cells disrupted the disulfide bond between DOX and PEG-b-PLL, causing nanoparticle decomposition and drug release. Although GSH is not a direct trigger for charge reversal, this pH and GSH dual-responsive charge-reversal drug delivery system enhances the selectivity for tumor cells while avoiding harm to other cells [59]. He et al. utilized reduction-sensitive disulfide bonds to modify hyaluronic acid to form ternary complexes (DPS complexes), which significantly increased its transfection efficiency and demonstrated its redox reactivity [60].

### 2.3. Enzyme-Responsive Charge-Reversal Systems

**Figure 3:**
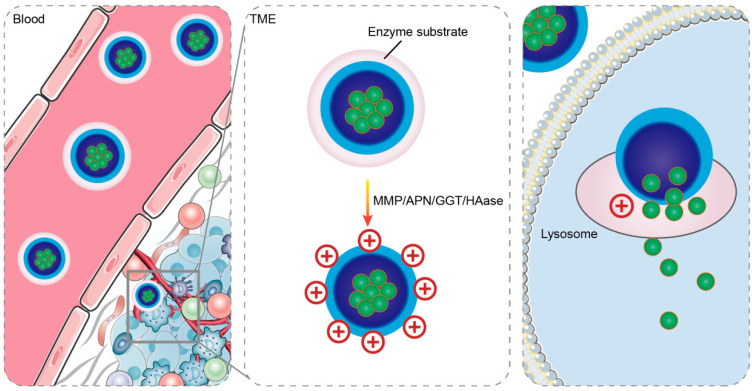
The outer substrate of the enzyme-responsive charge reversal system is hydrolyzed by specific enzymes in the TME, exposing the positively charged core. (Figure 3 was created with Blender 4.2.0).

Enzymes are the essential components that regulate cellular function and a range of physiological activities. Certain enzymes, such as matrix metalloproteinases (MMPs) [61], hyaluronidases (HAases) [62], γ-glutamyltransferases (GGTs) [63], aminopeptidases (APNs) [64], esterases [65], etc., are expressed more frequently in tumor tissues. By incorporating specific enzyme substrates into nanocarriers, NDDSs can respond to overexpressed enzymes both inside and outside the cell. The enzyme-responsive CRNDDS is vital in targeted tumor therapy and reducing side effects and their working principle is shown in Figure 3 below.

#### 2.3.1. MMP-Responsive Charge-Reversal System

MMPs are upregulated in most tumors, with MMP1, MMP9, MMP10, MMP11, and MMP13 being almost universally overexpressed in all cancers [66]. MMPs regulate pathways such as cell apoptosis, immune suppression, cell migration, and vascular migration [67]. Wu et al. synthesized McAL by conjugating DSPE with mPEG2000 through an MMP2-cleavable peptide linkage, creating the NanoValve that can be cleaved by MMP2. When exposed to the high expression of MMP2 in tumor cells, the PEG shell was removed, changing the zeta potential from negative to positive, promoting tumor cells uptake and preventing non-specific uptake by healthy cells [68]. Lang et al. developed a docetaxel (DTX)-loaded micelle (pDM) consisting of a pH-sensitive copolymer and an MMP9-responsive copolymer. When the pDM reached the tumor sites in 4T1 tumor-bearing mice, the PEG coating of the micelles was hydrolyzed under the action of MMP9, causing an increase in the concentration of DTX in tumor cells [69].

#### 2.3.2. APN-Responsive Charge-Reversal System

APN is a ZN2+-dependent membrane-bound metalloproteinase that preferentially cleaves neutral amino acids from the N-terminus of peptides [70]. The hydrolysis rate catalyzed by APN is related to the substrate structure [71]. APN is overexpressed in many infiltrating tumors and impacts cell proliferation, cancer invasion, capillary formation, etc. [72,73]. Thus, APN is considered an ideal enzyme for charge-reversal conjugates in tumor drug delivery. Sun et al.’s synthesized polymer-7-ethyl-10-hydroxycamptothecin (SN38) conjugates were with SN38-monomers (MMA-SN38). The conjugate remained neutral and stable in the blood circulation but generated a positive charge in tumor tissue due to APN-specific hydrolysis, leading to high drug accumulation and deep tumor penetration [74].

#### 2.3.3. GGT-Responsive Charge-Reversal System

GGT is an enzyme involved in the metabolism of glutathione, and it can transfer the gamma glutamyl group on glutathione to another peptide or amino acid, generating a primary amine. GGT plays an important role in the cell redox balance, proliferation and apoptosis balance, and it is overexpressed in some tumors [75]. The specific recognition and hydrolysis of γ-glutamylamides by GGT have been used to construct GGT-responsive charge-reversal polymers, enhancing drug penetration [76]. Dai et al. designed a GGT-responsive modular peptide with a negative surface charge in aqueous solution, limiting interaction with proteins and blood cells. In tumor tissue, the γ-glutamyl bond of the zwitterionic peptide was cleaved by the overexpressed GGT, and the charge reversal was achieved [77]. This GGT-triggered CRNDDS maintains a negative surface charge in normal tissue cells with low GGT concentrations, while reversing its surface charge in tumor tissues with high GGT concentrations. This system enhanced the tumor targeting and significantly improved the anti-tumor efficacy (68.48% vs. 24.07%, tumor inhibition rate, in contrast to free PTX) [78]. Zhou et al. found that highly hydrophobic polymer drug conjugates with a high camptothecin content exhibit higher GGT-reaction activity, resulting in faster cationization and enhanced cell internalization [79]. This insight aids in constructing GGT-responsive charge-reversal systems with higher tumor infiltration and also reveals the influence of hydrophilicity and hydrophobicity on enzyme-sensitive CRNDDSs.

#### 2.3.4. HAase-Responsive Charge-Reversal System

HAases are glycosidases that specifically degrade hyaluronic acid (HA). CD44, as its ligand, is overexpressed in many cancers and is associated with tumor progression, infiltration, and metastasis [80]. Researchers have developed various Haase-based CRNDDSs [81]. Yang et al. constructed a multiple stimuli-responsive nanoCRISPR (Must-nano) with a redox-sensitive core loading CRISPR/Cas9-targeting hypoxia-inducible factors-1α (HIF-1α) and a multiple-responsive shell anchored by chlorin e6 (Ce6). Must-nano can undergo charge reversal triggered by HAases and achieve site-specific release under the stimulation of redox signals [82].

## 3. Applications of Charge-Reversal Systems

### 3.1. Drug Delivery in Chemotherapy

Almost all the CRNDDSs remain neutral or negatively charged in the bloodstream, preventing interaction with normal cells and serum components. Upon reaching the TME, these systems are triggered by specific factors and converted into positive charges through various pathways. This transformation enhances the electrostatic interaction with negatively charged cell membranes and prevents lysosomal capture through the proton sponge effect [77,83,84]. Introducing carboxyl and tertiary amine groups into polyurethane and obtaining PUC-PUN co-assembled nano micelles through electrostatic interaction can create a system with acid sensitivity, achieving charge reversal from negative to positive in acidic environments. The use of PUC-PUN-1/DOX micelles for delivering DOX has been shown to improve its anti-cancer efficacy and safety [85]. The CRNDDS demonstrated precise tumor targeting ability, enhancing the killing of tumor cells while reducing the side effects of chemotherapy drugs due to its excellent biological safety [78,86]. In vitro experiments revealed a significant increase in cell apoptosis after JPD@L treatment, along with strong green fluorescence signals being detected in the cells. In B16-F10 mice, JPD@L showed the highest tumor inhibition rate (TIR) of 81.6%, significantly prolonging the survival time of B16-F10 tumor xenograft-bearing mice compared to other groups. These results indicate that the designed charge-reversal yolk-shell liposomes have efficient tumor-killing effects with low systemic toxicity [86]. As a carrier for chemotherapy drugs, it offers broad application prospects.

### 3.2. Dual- and Multi-Responsive CRNDDSs

Dual- and multi-responsive CRNDDSs have emerged as a new development trend in the field of CRNDDS research in recent years due to their ability to improve the accuracy of tumor targeting and the charge-reversal efficiency. They can respond to two or more stimulus signals, achieving a charge transition from negative to positive [87]. The common dual- and multi- responsive systems include pH/redox, pH/enzyme, and double pH system, and so on, which are listed in Table 1 below.

The pH difference is the most common distinguishing factor between the TME and the normal blood environment. The high concentration of GSH in tumor tissue is another widely targeted characteristic. It is used in redox-reactive charge-reversal and size-reduction NDDSs, as GSH can cleave the disulfide bonds in polymers [92]. Disulfide-containing hyperbranched MeO-PEG-b-(NIPAAm-co-PBAE) polymeric nanoparticles have been synthesized, which switch to a positive charge in an acidic TME and then release drugs rapidly due to the size reduction caused by the high concentration of GSH in tumor cells [44]. Tao et al. designed a kind of acid/reduction dual-sensitive amphiphilic graft polyurethane with folic acid and detachable poly(ethylene glycol) (FA-PUSS-gimi-mPEG). When the micelle entered the acidic TME, the benzoimide bond underwent cleavage to achieve surface charge reversal, while high concentrations of GSH increased the drug release rate [31].

### 3.3. Gene-Delivery Therapy

Gene therapy is an important component of tumor treatment, with viruses widely used as gene carriers [93]. However, viral vectors pose issues such as immunogenicity, tumorigenicity, and many other side effects. Therefore, developing gene vectors with high transfection efficiency and low cytotoxicity is crucial for gene therapy. In recent years, charge-reversal nanosystems have been widely applied in gene delivery [94,95].

Researchers encapsulated the coding plasmid (pDNA) in lipid nanoparticles (LNPs), modified with fish sperm protein coupled with palmitic acid on the surface, and coated it with sodium tripolyphosphate (TPP) on the outermost layer. Compared with pDNA alone, the transfection efficiency was significantly improved, and human alveolar epithelial cells (A549) showed good tolerance [96]. Zhang et al. designed a polymeric nanocarrier (BTIL) composed of B-PDEAEA/DNA polyplex-based cores and IR780-loaded liposome coatings. Under low-intensity ultrasound irradiation, IR780 generated high concentrations of ROS, causing ROS-responsive charge reversal in the B-PDEAEA polymer, leading to intracellular gene release and efficient gene transfection. BTIL maintains stable circulation in the bloodstream for a long time and has high efficiency in terms of gene delivery with minimal side effects [97]. This research offers new ideas for non-viral gene-delivery vectors.

### 3.4. Chemo-Photothermal Therapy

In recent years, photothermal therapy (PTT) has been widely used in tumor treatment. It refers to the application of materials with high photothermal conversion efficiency to convert light energy into heat energy under external light sources, thereby killing tumor cells [98,99].

Chemo-photothermal therapy has become a common strategy for tumor treatment. Chemotherapy can compensate for the shortcomings of tumor recurrence and inability to eradicate tumors in photothermal therapy, while photothermal therapy can also increase the chemotherapy toxicity. CRNDDSs have shown promising application in this field [100,101].

Wang et al. developed a core–shell structured polymer nanoparticle (MPPS@IR825/DTX NPs), in which the DMMA-modified polyethylene glycol shell transformed into a positive charge in an acidic environment, enhancing tumor permeability. Under near-infrared laser irradiation, polymer nanoparticles were rapidly dissociated, and the encapsulated photosensitizer (IR825) converted light energy into heat, improving the release efficiency of DTX during local hyperthermia [102]. Kang et al. encapsulated the chemotherapy drug gemcitabine (Gem) and photosensitizer IR1048 in a GSH-responsive polymer (SGP), followed by an enzyme-responsive HA coating to form a dual-cascade responsive NP (sNP@G/IR) [103]. Deng and Li have, respectively, constructed two types of nanomedicine systems based on ZnO@CuS and black phosphorus nanosheets (BP NSs). Both systems used dimethylformamide bonds for pH-responsive functionality, along with photothermal properties to kill tumor cells and increase drug penetration into the tumor [104,105].

### 3.5. Contrast Agents Delivery

Imaging techniques, such as computed tomography (CT) and magnetic resonance imaging (MRI), are crucial for tumor screening, diagnosis, and evaluation. Contrast agents improve the diagnostic accuracy by increasing the contrast [106,107]. There is an urgent need to develop nanocarriers that can specifically deliver contrast agents to tumor tissue.

Feng et al. designed a TME-responsive nanocarrier (MB@MSP) using mesoporous silica nanoparticles (MSNs) and Fe_3_O_4_ as the core, with the photosensitizer methylene blue (MB) encapsulated in the mesopores of the MSNs. MMP2-degradable peptide PDPPA-1 was coupled with the core via disulfide bonds. In the tumor matrix, highly expressed MMP2 cleaves PDPPA-1 and high GSH concentrations cause disulfide bond cleavage, resulting in a size reduction and charge reversal, promoting the penetration of nanocarriers into tumors. MB@MSP enables MR and μCT imaging in vivo and induces the death of immunogenic tumor cells [108]. He et al. co-loaded gadolinium oxide (Gd_2_O_3_) and DOX into MSNs to form hybrid nanoparticles (Gd_2_O_3_@MSNs), coating the surface with pH-responsive polyelectrolytes. Charge reversal occurred in acidic environments, achieving contrast agent (Gd_2_O_3_) delivery simultaneously with drug delivery [109].

### 3.6. Other Treatment Modalities Combined with CRNDDSs

In malignant tumors, although chemotherapy can produce cytotoxic effects on tumor cells, its actual efficacy is affected by immune suppression and immune escape [110,111]. Therefore, immunotherapy combined with chemotherapy has become an emerging method for treating cancer, and the development of size-/charge-variable nanocarriers for drug delivery can achieve good delivery efficiency while overcoming biological barriers. Immune checkpoint blockade (ICB) therapy is a commonly used anti-tumor immunotherapy. The combination of PD-1 and PD-L1 can transmit immunosuppressive signals, prevent T cell activation, and promote immune escape of tumor cells. Therefore, blocking the PD-1/PD-L1 axis has become one of the effective strategies for activating T cell-mediated anti-tumor immune responses [112]. Liu et al. constructed a yolk-shell liposome co-loaded with JQ1 and DOX, which can achieve rapid changes in the zeta potential from −4.90 to +2.35 in acidic environments, thereby achieving efficient cellular internalization. By inhibiting the PD-L1 pathway and DOX-induced immunogenic cell death (ICD) through JQ1, efficient chemoimmunotherapy was ultimately achieved [86]. Table 2 below shows the applications of combining ICB therapy with CRNDDSs.

Many studies have shown that tumor-associated macrophages (TAMs) are an important component of the tumor immune microenvironment and can identify two main macrophage phenotypes M1 and M2 with different functions [117]. TAMs typically undergo macrophage polarization during tumorigenesis, transitioning from the M1 anti-tumor phenotype to the M2 pro-tumor phenotype [118]. M2-like macrophages play a role in angiogenesis, immune suppression, and promoting metastasis in the TME [119]. Therefore, targeting TAMs is an important strategy in tumor immunotherapy. The following Table 3 summarizes the CRNDDSs targeting TAMs.

## 4. Limitations of CRNDDSs

As a carrier for drug delivery in the human body, the biocompatibility and toxicity of nanocarriers for constructing CRNDDSs must be strictly monitored and controlled, especially inorganic materials such as carbon nanotubes and mesoporous silica nanoparticles (MSNs). There are articles revealing that the types and quantities of metal impurities, the lengths and types of carbon nanotubes, the presence of solubilizers, and the functionalization of carbon nanotubes can all affect their toxicity [123]. These factors need to be carefully considered when using carbon nanotubes to prepare CRNDDSs. In addition, carbon nanotubes also have problems such as poor solubility, low biodegradability and dispersibility, indicating that this material urgently needs to be optimized or replaced [124]. The toxicity of MSNs is influenced by factors such as the particle shape, size, pore size, and synthesis method. Studies have shown that MSNs with a particle size of 55 nm have lower cytotoxicity than other particle sizes [125]. When researchers use an MSN as a carrier, they need to consider both its surface modification to determine biological distribution and the characteristics of the MSN to reduce cell toxicity.

For dendritic polymers, their toxicity is mainly influenced by their size. Poly(amidoamine) dendrimers (PAMAM) can be divided into many generations, where the sizes of the PAMAM from Generation 0 to 5 (G0–G5) are all less than 5.7 nm and they are mainly metabolized in the kidneys with low toxicity, while the sizes of PAMAM from Generation 6 to 9 (G6–G9) are all greater than 7.2 nm and they are metabolized in lymph nodes or liver with high toxicity [126].

There are concerns regarding the triggering factors of charge reversal: the TME exhibits heterogeneity, and there may be differences in the pH values and specific enzyme concentrations in the TMEs of different types of tumors, which may affect the charge reversal of pH-responsive and enzyme-responsive CRNDDSs in the TME [127]. Most photoresponsive CRNDDSs require near-infrared (NIR) light stimulation. Although NIR light has a deeper penetration depth than visible and ultraviolet light, there may be limitations in its penetration effect for thicker skin and muscle tissues [128]. At present, most research is conducted using mice implanted with tumors subcutaneously, and the efficacy in treating human tumors is still unknown [129]. Therefore, tumors suitable for using photoresponsive CRNDDSs should be selected to apply this type of CRNDDS, ensuring the penetration effect of light.

In recent years, the heterogeneity of the TME has gained increasing attention, which plays an important role in the treatment efficacy and the occurrence of drug resistance. Different tumors possess distinct tumor immune microenvironments, necessitating targeted drug development to achieve specific treatment for these varied immune microenvironments. Obviously, CRNDDSs should not be confined to the current known stimulating factors, and more specific triggering conditions are waiting to be explored.

## 5. Conclusions

The CRNDDSs have the ability to respond to various stimuli, such as acid, redox, and enzymes, so that they can achieve a negative to positive charge conversion. Therefore, they can adapt well to the characteristics of the TME and achieve specific drug delivery. Consequently, CRNDDSs have gained widespread application in cancer treatment.

In this review, we summarized the recent advancements in CRNDDSs in terms of tumor treatment over the past two years, categorizing their working principles and advantages of action based on different stimuli. Although studies have shown that CRNDDSs exhibit higher killing efficiency and precision compared to standalone drugs, most validations have only been conducted in vitro, and the biological safety and efficacy of CRNDDSs in vivo have not been thoroughly explored. With the continuous development of new nanomaterials, the sensitivity of CRNDDSs to factors such as pH, redox, and enzymes has been ensured, which improves the delivery efficiency and specificity of drugs. However, further research is needed to confirm the in vivo safety and efficacy of these systems to advance toward clinical application.

## Figures and Tables

**Table 1:** Common dual- and multi-responsive CRNDDSs ^1^.

Stimulus	Responsive Component	Reference
pH/ROS	2,3-dimethylmaleic anhydride (DMA); thioketal (TK)	[88]
pH/GSH	tertiary amine groups; disulfide bond	[44]
	benzoic-imine bond; disulfide bond	[31]
ROS/pH	thioketal (TK); PBAE	[43]
pH/pH	β-carboxylic amide; Schiff base	[89]
	Histidine; hexahydrobenzoic amide	[90]
pH/MMP-2	PEG-His; MMP-2 response peptide (PLGVRKLVFF)	[48]
pH/MMP-9	poly((1,4-butanediol)-diacrylate-β-N,N-diisopropylethyl-enediamine)-polyethyleneimine (BD-PEI); poly((1,4-butanediol)-diacrylate-β-N,N-diisopropylethylenediamine)-PLG-PEG (BD-PLG-PEG)	[69]
Thermal/pH/redox	poly(N-isopropylacrylamide) (PNIPAM); cystamine; phosphate groups	[91]

^1^ The table displays CRNDDSs of different stimulus types and shows their respective response to stimuli.

**Table 2:** Applications of combining ICB therapy with CRNDDSs ^2^.

Treatment Modalities	Charge Reversal Stimulus	Advantages	Reference
Immunotherapy	pH/redox	Programmable drug delivery and chemotherapy-enhanced indoleamine 2,3-dioxygenase (IDO) immunotherapy strategy	[113]
Immunotherapy	ROS	Starvation/oxidation-integrated IDO blockade immunotherapy	[114]
Immunotherapy and PDT	MMP2/GSH	PDT combined ICB therapy, performing MR and μCT imaging in vivo	[108]
Immunotherapy	pH	Excellent synergistic inhibitory effect on B16-F10 tumor growth	[86]
Immunotherapy	photo	Achieving photo-enhanced intracellular delivery of Ce6 and iFSP1 to induce synergistic ferroptosis, thereby leading to ICD	[115]
Immunotherapy and PDT	pH/MMP2	Improving the immuno-photodynamic therapeutic effect and alleviating the immune-related adverse events	[116]

^2^ The table shows a series of application examples that combine immunotherapy and photodynamic therapy with CRNDDSs, utilizing ICB therapy.

**Table 3:** Examples of CRNDDSs targeting TAMs ^3^.

Treatment Modalities	Charge Reversal Stimulus	Effect	Advantages	Limits	Reference
Immunotherapy	pH	TAMs repolarized to the M1 type	The first combination of charge-reversal polymers and OMVs used to realize a separation function	Further research is needed on the internal mechanism of charge-reversal polymer-induced vesicle destruction	[25]
Immunotherapy	pH	Re-educate M2-TAMs to the M1 phenotype	Delivering siVEGF and siPIGF to both M2-TAMs and breast cancer cells for synergistic anti-tumor immunotherapy		[120]
Immunotherapy	pH	Repolarizing M2-TAMs and inducing the ICD	Demonstrating the best anti-tumor effect in vivo and suppressing MDSCs and Tregs to further reconstruct the ITM		[110]
Immunotherapy	pH	Induced cell cycle arrest, enhanced TAM repolarization, inhibited Treg cell function	Utilizing the novel triple-interlocked combination therapy on chemotherapy, immunotherapy, and chemoimmunotherapy		[121]
Immunotherapy	pH	Inhibiting immune cell recruitment and pro-inflammatory factors stimulus	Emerging strategies of immunotherapy for the prevention and treatment of atherosclerosis		[122]

^3^ The table shows the applications of CRNDDSs targeting TAMs, summarizing their stimulating factors, effects, advantages and disadvantages.

## Data Availability

Not applicable.
